# brain-coX: investigating and visualising gene co-expression in seven human brain transcriptomic datasets

**DOI:** 10.1186/s13073-017-0444-y

**Published:** 2017-06-08

**Authors:** Saskia Freytag, Rosemary Burgess, Karen L. Oliver, Melanie Bahlo

**Affiliations:** 1 Population Health and Immunity Divison, The Walter and Eliza Hall Institute of Medical Research, 1G Royale Parade, 3052 Parkville, Australia; 2 Department of Medical Biology, University of Melbourne, 1G Royale Parade, 3052 Parkville, Australia; 3 Epilepsy Research Centre, Department of Medicine, Austin Health, University of Melbourne, 245 Burgundy Street, 3084 Heidelberg, Australia; 4 School of Mathematics and Statistics, University of Melbourne, 3010 Parkville, Australia

## Abstract

**Background:**

The pathogenesis of neurological and mental health disorders often involves multiple genes, complex interactions, as well as brain- and development-specific biological mechanisms. These characteristics make identification of disease genes for such disorders challenging, as conventional prioritisation tools are not specifically tailored to deal with the complexity of the human brain. Thus, we developed a novel web-application—brain-coX—that offers gene prioritisation with accompanying visualisations based on seven gene expression datasets in the post-mortem human brain, the largest such resource ever assembled.

**Results:**

We tested whether our tool can correctly prioritise known genes from 37 brain-specific KEGG pathways and 17 psychiatric conditions. We achieved average sensitivity of nearly 50%, at the same time reaching a specificity of approximately 75%. We also compared brain-coX’s performance to that of its main competitors, Endeavour and ToppGene, focusing on the ability to discover novel associations. Using a subset of the curated SFARI autism gene collection we show that brain-coX’s prioritisations are most similar to SFARI’s own curated gene classifications.

**Conclusions:**

brain-coX is the first prioritisation and visualisation web-tool targeted to the human brain and can be freely accessed via http://shiny.bioinf.wehi.edu.au/freytag.s/.

**Electronic supplementary material:**

The online version of this article (doi:10.1186/s13073-017-0444-y) contains supplementary material, which is available to authorized users.

## Background

The World Health Organization estimates that around 450 million people worldwide suffer from mental or neurological conditions, placing these disorders at the top of the list of global disease burdens [1]. A better understanding of biochemical and morphological abnormalities in affected brains can help alleviate this burden. Next to non-invasive neuroimaging and post-mortem histological analysis, the identification of genes involved in the pathogenesis of these disorders is the most promising avenue to improve our knowledge and consequently develop better diagnostics, treatments and targeted therapeutics [2].

In recent years, genome-wide association studies, as well as high-throughput sequencing of families, have identified hundreds of variants located in, or near, coding regions compellingly statistically associated with mental and neurological disorders [3, 4]. For many of the variants, however, the functional alleles and mechanisms that give rise or contribute to these disorders remain elusive. For example, while more than 100 loci are associated with schizophrenia, few genes have been implicated in the biological process underlying this genetically complex disease [5]. Similarly, about 1000 copy number variants and rare and common variants have been found to be associated with autism [6], but little is known about how they confer risk.

Designing and performing scientific experiments to generate functional evidence for the involvement of a gene in a mental or neurological disorder is often challenging. The cost of such experiments is typically high, in particular when human brain tissue is necessary. Furthermore, because of the large number of putative disease genes only a subset of genes can be followed up. Fortunately, computational methods as well as visualisation techniques exist that can help to prioritise which candidate genes to pursue with such methods. These methods are referred to as in silico prioritisation. These methods typically rely on knowledge collected in genomic databases, such as the Online Mendelian Inhertiance in Man (OMIM) database [7], as well as gene expression data from healthy individuals [8, 9]. Popular examples of computational methods offering in silico prioritisation include Endeavour [10] and ToppGene [11]. One of the most frequently employed visualisation tools for gene networks is String [12].

Both in silico prioritisation and gene network visualisation tools have been successfully applied to many diseases [13, 14]. Nevertheless, most tools are biased towards what is already known due to their reliance on genomic databases and literature searches via databases such as PubMed [15]. Because of this known bias some tools also integrate gene expression data from healthy individuals to implicate disease pathways discovered de novo from the data. However, such gene expression data are usually generated from easily obtained sources, such as blood or lymphocytes, and thus may not reflect the pathways in the relevant disease tissue [16, 17]. Gene expression in the brain is uniquely different from other tissues, reflecting the complex biological processes in the brain [18]. Leveraging such brain-specific gene signatures has indeed been shown to be beneficial in uncovering disease genes for epileptic encephalopathies [19]. Furthermore, many available tools do not take into account that gene expression varies considerably over the course of an individual’s development, especially in the brain. For example, in the human brain, Kang et al. [20] observed that gene expression is regulated to a large degree temporally and only to a lesser extent spatially.

Very few tools offer both in silico prioritisation and gene network visualisation, which hinders interpretation and design of functional downstream analysis [8] (one notable exception is the downloadable application NETBAG [21]). brain-coX is a novel web-application focusing on gene prioritisation and exploration of gene networks for diseases that originate in human brain tissue. Unlike any of the existing tools, brain-coX’s prioritisations are based solely on brain expression data, making use of up to seven available large datasets measuring gene expression in the developing and ageing human brain. These datasets were processed and cleaned in a homogeneous manner, ensuring maximal reproducibility of results across datasets. To our knowledge this is the first time results from these seven precious brain expression datasets are directly comparable, within one resource. Besides prioritisations, brain-coX also allows users to investigate pathway membership and to explore changes in gene networks throughout brain development via interactive visualisations. Such temporal changes in gene networks have been hypothesized to play a key role in many neurological and mental disorders, with many such disorders showing distinct ages of onset. Finally, we designed brain-coX to be user-friendly and easily accessible through a website to facilitate use by researchers who are not comfortable with command line tools.

## Implementation

### Datasets

#### Dataset descriptions

We downloaded seven published and publicly available datasets of gene expression from post-mortem human brain tissue samples (Table 1). For six out of seven datasets, samples were collected from individuals deemed to be normal with respect to mental and neurological disorders. The Hernandez et al. dataset [22] contains some individuals with unknown disease status. Datasets differ widely with regards to age range of individuals, number of individuals and number of samples as well as tissue types collected from each brain. To cater for this, brain-coX allows the user to select any combination of these datasets. Furthermore, users are able to further subset data by specifying developmental periods of interest. To this end, the individuals contributing samples were assigned to 15 different developmental periods according to their age at death (Table 2). This option facilitates targeted prioritisation and gene exploration for specific diseases. An example would be a disease with onset in childhood where a focus on brain samples from this time period are likely to be much more informative than samples from other time periods.

**Table 1:** Key features of the seven different gene expression datasets of the developing and ageing brains

Gene expression resource/publication	Platform	Number of individuals	Average number of arrays per brain	Number of time periods
Hawrylycz et al. [31]	Agilent	10	406	2
Miller et al. [44]	Agilent	4	328	2
Colantuoni et al. [45]	Custom	266	1	11
Kang et al. [20]	Affymetrix	57	24	15
Hernandez et al.^a^ [22]	Illumina	397	2	8
Trabzuni et al. [46]	Affymetrix	134	9	4
Zhang et al. [47]	Agilent	101	3	3

^a^This dataset contains some individuals who were not normal with respect to neurological and mental health disorders

**Table 2:** Fifteen developmental periods of the human brain as defined by Kang et al.

Period	Description	Age range
1	Embryonic	4–8 PCW
2	Early fetal	8–10 PCW
3	Early fetal	10–13 PCW
4	Early mid-fetal	13–16 PCW
5	Early mid-fetal	16–19 PCW
6	Late mid-fetal	19–24 PCW
7	Late fetal	24–18 PCW
8	Neonatal and early infancy	Birth to 6 M
9	Late infancy	6 M–1 Y
10	Early childhood	1–6 Y
11	Middle and late childhood	6–12 Y
12	Adolescence	12–20 Y
13	Young adulthood	20–40 Y
14	Middle adulthood	40–60 Y
15	Late adulthood	60+ Y

*M* months, *PCW* post-conception weeks, *Y* years

#### Pre-processing and data cleaning

Different experimental protocols and microarray platforms were used in the generation of these seven datasets, leading to diverse sources of unwanted biological and technical variation. Thus, homogeneous pre-processing and data cleaning are vital in order to ensure that these heterogeneous datasets are comparable [23]. During pre-processing each sample was assessed for its quality and samples with poor quality spot plots, unusual plots of log-intensity ratios versus log-intensity averages or abnormal gene expression distributions were excluded. After pre-processing, each dataset is treated separately with one of two implemented cleaning strategies. Users can choose between conventional background correction [24] in combination with quantile normalisation [25] or removal of unwanted variation (RUV) [26], a data-driven approach. Unlike most other cleaning approaches, such as ComBat [27], these two approaches do not require meta data (i.e. batches, laboratory, etc.) on the samples, which in most datasets was partially or not at all available.

RUV removes unwanted variation in an adaptive manner with the help of negative control genes. Such genes are affected by unwanted variation, but crucially not by the biological variation of interest. The default setting in brain-coX is to take all house-keeping genes as negative control genes, but these can also be empirically chosen. When unwanted variation and biological variation of interest are correlated with each other, RUV removes biological signal. In order to account for such correlation to a degree, brain-coX applies a version of RUV with a regularization parameter, as previously described [28]. To prevent further removal of biological variation of interest, brain-coX also excludes known disease genes, candidates and further genes specified by the user from being negative control genes. This method has been demonstrated to reliably recover gene–gene correlations, which form the basis of in silico prioritisation and network visualisations. Furthermore, its application to a subset of the brain datasets demonstrated improved reproducibility across datasets compared to other cleaning strategies [28]. Here, we also demonstrate increased accuracy prioritising known pathway genes compared to background correction and quantile normalisation (Additional file 1: Figures S1 and S2). Furthermore, RUV also considerably reduces differences between datasets. When the seven datasets are combined, differences between the datasets are noticeably reduced and the remaining clustering can be attributed to developmental differences rather than data sources (compare Additional file 1: Figures S3, S4, S5 and S6).

### Prioritisation

brain-coX prioritises user-supplied candidate genes via the guilt-by-association principle [29]. This principle assumes that the most promising candidate genes will be the ones that are associated with genes already known to be involved in the disease. Such candidates are likely to be part of the same biological network(s) that, when disrupted, lead to the development of the disease. The reliance on this principle means that the user is required to supply already known disease genes in order to prioritise their candidate genes. This approach has been shown to work well in many neurodevelopmental disorders where the list of discovered genes continues to grow. However, it should be noted that the guilt-by-association principle assumes that all disease genes fall into a small number of convergent pathways. If this is not the case, discovery of new disease genes is unlikely.

As gene prioritisation is the focus of brain-coX, we implemented an improved version of a prioritisation strategy, BrainGEP, proposed by Oliver et al. [30]. Using a retrospective analysis, they were able to assess the validity of their prioritisations. In Oliver et al. 179 putative epileptic encephalopathy candidates were examined, of which 19 had been prioritised in 2013. They found that six candidates had since been confirmed, of which their prioritisation had predicted five [19]. This result is based on the use of only the Allen Human Brain Atlas expression dataset [31] while brain-coX prioritises candidates on up to seven datasets simultaneously and compares the results. Furthermore, brain-coX employs a different weighting of the simple Pearson correlation to BrainGEP. In brain-coX, correlations are weighted by the inverse of the number of samples contributed by the respective donor in order to take into account dependencies.

The prioritisation can be summarised in three steps, which are conducted on each of the seven datasets available in brain-coX separately (also compare Additional file 1: Figure S7).

#### Step 1: Determination of background correlation

A random set of genes, the size of the candidate gene set, is selected. The absolute weighted correlation between these random genes and the known disease genes is then calculated. For each gene only the maximum absolute correlation is retained. This step is repeated 1000 times.

#### Step 2: Determination of correlation threshold

The user selects a proportion of non-disease genes allowed to be prioritised. The 1000 repeats of step 1 can then be used to determine the threshold for the absolute correlation, ensuring the user-selected proportion of random genes gets prioritised on average. Note that this proportion is an overestimate as not all randomly selected genes will be truly biologically independent of the disease genes.

#### Step 3: Prioritisation

Finally, brain-coX calculates the correlation between the candidate genes and the disease genes. With the cutoff for the absolute correlation established, candidate genes that have a maximum absolute correlation with any disease gene greater than the cutoff are prioritised.

As prioritisation is performed separately in every selected dataset, the number of datasets a candidate gene is prioritised in can thereby be seen as an indicator of the likelihood that the candidate gene is truly associated with the disease genes. Candidate genes can be further ranked by their sum of all absolute correlations, above the calculated threshold with any known disease gene.

### Exploration through visualisation

#### Network visualisation

brain-coX has extensive visualisation options that allow an intuitive understanding of the prioritisation results. The tool offers two types of network visualisations. The first uses the datasets separately and is designed to highlight the effect of individual datasets on prioritisation results. The user can also interactively assess how different choices of parameters, such as the proportion threshold, influence the results. For the second visualisation, datasets were combined and the user can investigate different clustering algorithms on the gene–gene correlation heatmap. Furthermore, users also have the option to explore partial correlations, which control for indirect interactions between genes.

#### Investigating the effect of brain development

Gene expression networks are known to alter in response to environmental cues and factors during development [32]. Users can explore such changes with heatmaps of gene–gene correlations estimated for each time period independently. They can also focus on the changes occurring in gene regulation in the normal human brain between sets of time periods. We believe that this feature in particular may help to pinpoint disease-relevant developmental mechanisms that are disrupted in patients. We have included a case study in Additional file 1 to explain the use of these features in learning more about candidate genes.

### Interface

The graphical user interface of brain-coX was built using shiny [33]. Like other shiny applications, brain-coX leverages R [34] and Bioconductor [35] resources for the underlying calculations and plot output. Due to shiny’s inherent reactive programming framework, output is only updated when the user changes the settings or instigates a new query. There are two ways to run brain-coX: firstly, it can be accessed through our web-server (http://shiny.bioinf.wehi.edu.au/freytag.s/), requiring no further programmes to be installed; secondly, it is available as a local version once the software is downloaded and installed. The latter has the advantage of reducing computational time and not being limited by the host web-server’s current load. However, this requires a recent version of R and several additional R and Bioconductor packages on which several steps rely. Furthermore, executing the application requires basic knowledge of R.

For a detailed example explaining the use of brain-coX to identify zinc transporter genes that may play a role in febrile seizures see Additional file 1 and Additional files 2, 3 and 4 for the associated data.

## Results

### Statistical benchmarking

We followed the leave-one-out cross-validation approach described by Aerts et al. [10]. In this approach one gene is deleted from the known set of genes and termed the “defector” gene. The ability to prioritise this gene in a list of 99 other candidates, made up of random genes not known to be associated, determines the accuracy. Unlike Aerts et al., we used two different types of known gene sets that will reflect a spectrum of networks, with some gene sets likely to be connected within one network and other gene sets showing very little connection. The former gene sets will do well with our approach, the latter will not. The first set of genes consists of 37 KEGG pathways [36] which function in the human brain as judged by keywords search (Additional file 1: Table S2). The second set of genes was automatically mined from the PsyGeNet database [37], a resource that stores genes associated with psychiatric diseases (for a full list see Additional file 1: Table S3). This set contained 17 diseases, such as major affective disorder and anhedonia, and their known genes.

For the prioritisation approach implemented in brain-coX with RUV normalisation, we also examined the effect of using multiple datasets. To do this we defined a successful prioritisation as a gene prioritised in at least k datasets. We then found the average number of false positives and true negatives as well as the number of false negatives and true positives in every pathway for each k. This allowed us to calculate the specificity, sensitivity, precision and negative prediction value. In this context, sensitivity thus quantifies the prioritisation approach’s ability to correctly prioritise the “defector gene”. Similarly, specificity allows judging an approach’s tendency to prioritise random genes that are not involved in the disease. Generally, a prioritisation approach with high specificity is preferred, as this reduces costs involved in the follow-up of false candidate genes.

**Fig. 1:**
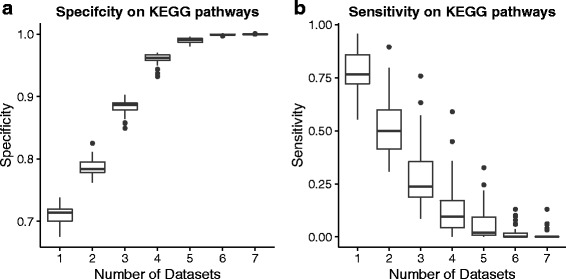
Accuracy of brain-coX in predicting KEGG pathways. The displayed accuracy measures were generated from leave-one-out cross-validation using 37 KEGG pathways that function in the human brain. We also examine the effect of requiring a gene to be prioritised in multiple datasets on the accuracy measures. **a** Specificity of brain-coX prioritisation approach. **b** Sensitivity of the brain-coX prioritisation approach

**Fig. 2:**
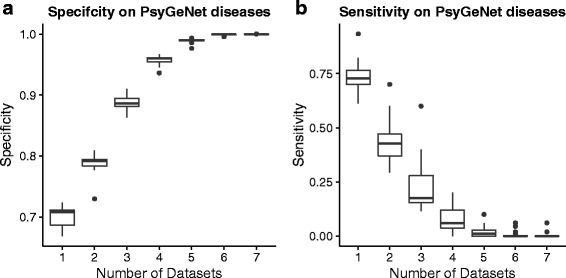
Accuracy of brain-coX in predicting disease genes in PsyGeNet. The displayed accuracy measures were generated from leave-one-out cross-validation using 17 PsyGeNet diseases that function in the human brain. We also examine the effect of requiring a gene to be prioritised in multiple datasets on the accuracy measures. **a** Specificity of brain-coX prioritisation approach. **b** Sensitivity of the brain-coX prioritisation approach

Our prioritisation approach (at 20% correlation threshold) has mean specificity above 0.70 for any one of the brain array resources, or combinations thereof, for both the KEGG and PsyGeNet set of known genes (compare Figs. 1 and 2). Sensitivity rapidly decreases with the number of datasets required to prioritise the “defector” genes. Requiring a gene to be prioritised in at least two datasets seems to result in the best trade-off between specificity and sensitivity of the method when precision and negative prediction value (Additional file 1: Figures S8 and S9) are also considered. It is interesting to note that the sensitivity values for the PsyGeNet sets are only slightly below those found for the KEGG gene sets. This suggests that gene expression networks can be utilized for the identification of disease genes in psychiatric diseases much like for the construction of pathways. A large gene co-expression network functionally related to synaptic transmission and recently identified to be differentially regulated in schizophrenia is further testament to this [38]. It also suggests the utility of brain-coX for the interpretation of neuropsychiatric GWAS results.

#### Benchmarking of different cleaning strategies

We also compared prioritisation accuracy for the two different normalisation techniques implemented in brain-coX, RUV and background correction combined with quantile normalisation. We determined sensitivity and specificity from leave-one-out cross-validation on the 37 KEGG pathways as described previously. Using these pathways, sensitivity appears to be roughly similar for the two approaches (Additional file 1: Figure S10). There are statistically significant gains in specificity (*t*-test, *t*-statistics = 8.49, *p* value = 7.73e-12) when using RUV normalisation compared to conventional normalisation with background-correction and quantile normalisation. Our previous work published on RUV normalisation applied to correlations indicated greater reproducibility of prioritisation results between datasets for epileptic encephalopathy candidates when using the RUV approach compared to the conventional approach [28].

### Comparison with other web-applications

Comparing the performance of brain-coX with other prioritisation web-tools is challenging. Most web-based prioritisation tools already integrate databases such as OMIM, DisGeNet [39] and KEGG in order to improve their performance. Thus, neither the KEGG pathways nor the PsyGeNet disease genes should be used to compare performance of different tools, as such a comparison would be heavily biased in favour of web-tools that make use of these resources. Moreover, such a comparison does not reflect the real user case where these tools are being used to discover novel disease genes and pathways.

We compare brain-coX to two competing web-based prioritisation approaches, Endeavour and ToppGene. Both approaches rank candidate genes with the help of known disease genes. We chose not to compare the performance of brain-coX to prioritisation approaches that do not offer web interfaces. We acknowledge that such tools could potentially offer superior performance, but they cannot be used without some knowledge of programming. One example is Weighted Gene Co-Expression Network Analysis (WGCNA) [14], the most commonly used network construction tool. While this tool requires extensive optimization of parameters, it allows temporal effects to be taken into account. We did investigate how the performance of WGCNA compares to our approach for a subset of 37 KEGG pathways described above. We found that brain-coX performs better, distinguishing between genuine pathway genes and random genes, than WGCNA (Additional file 1). Note that we also do not compare brain-coX to prioritisation tools specialised for only one disease, such as the algorithm Detecting Association With Network (DAWN) for autism [6], which uses expert knowledge. Such tools might outperform brain-coX. However, for most neurological and mental diseases specialised tools do not yet exist.

In order to overcome the bias in the leave-one-out cross-validation studies with known pathways, we also investigated a set of genes that a priori is expected to harbour several true positive genes. We made use of the curated Simons Foundation Autism Research Initiative (SFARI) Gene database [40], which is expected to contain likely disease-causing autism genes. These genes are furthermore ranked in terms of confidence, providing a further source of data for validation. The 826 genes in the database are scored from 1 to 6; each value indicating a category. The first category includes high-confidence autism genes and the last category contains genes that are currently not supported by any evidence for their involvement in autism.

To compare the performance of Endeavour, ToppGene and brain-coX, we used the 17 genes in the high-confidence group (category 1) as our known autism genes. We designated 340 genes in categories 2 to 6 that were not associated with autism in DisGeNet as candidate autism genes. We restricted brain-coX to only use samples collected from early mid-fetal development to early childhood (periods 4–10). Autism typically manifests in the first or second year of life [41], but there is robust evidence for the involvement of early mid-fetal development in this disease [42]. We used both ToppGene and Endeavour largely with default settings; however, we excluded the use of BLAST Annotation for Endeavour to ensure completion of the prioritisation with large sets of candidates. brain-coX prioritised 222 genes at 20% threshold in at least one dataset, while ToppGene prioritised all candidates and Endeavour prioritised only two genes according to the associated *p* values.

**Fig. 3:**
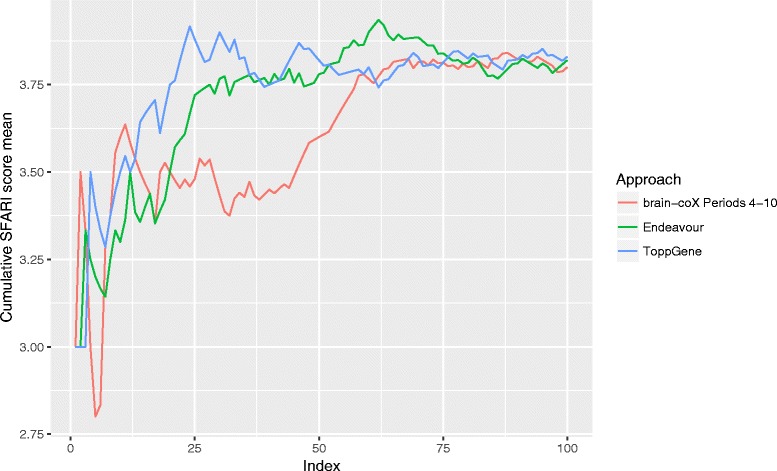
Cumulative mean score for SAFRI candidate genes. We prioritised 340 genes in the SAFRI database for autism with three different prioritisation approaches given 17 known autism genes. For the first 100 prioritised genes of each method we calculated the cumulative mean of the respective SFARI scores (2–6). Lower scores indicate genes that are more likely to be involved in autism

In order to assess the quality of the prioritisation of each approach we examined the cumulative average of the gene score assigned by SFARI Gene to the first 100 ranked genes (compare Fig. 3). Thus, a low score indicates good performance. The cumulative average score for the genes prioritised by brain-coX was either equal to or lower than that with either Endeavour or ToppGene. While there was not much difference between the different prioritisation approaches for the first 20 genes, brain-coX yielded considerably lower scores for genes ranked from 21 to 59. This indicates that brain-coX prioritises genes at least until rank 50 that have more support for involvement in autism than the other tools.

The SFARI Gene database relies on the research community for the collection of autism genes and is thus expected to be incomplete. Furthermore, it is governed by its own set of annotation rules, which may create certain biases. Because of this, we conducted a second performance analysis which was identical to the first in all but the choice of candidate genes. Here, the candidate genes were chosen from an association analysis conducted by Sanders et al. [43] on data from the Autism Genome Project, the Autism Sequencing Consortium and the Simons Simplex Collection. In total, we used 41 genes that reached significance (false discovery rate (FDR) ≤0.1) for association with autism and were not found in DisGeNet.

To compare the approaches we examined the Spearman correlation between the prioritisation rank and the FDR value of association with autism for all prioritised genes. Given the wealth of data used by Sanders et al., the FDR value can be viewed as a proxy of the likelihood of true involvement in autism. The Spearman correlation for brain-coX was the highest at 0.259 (0.135 for Endeavour, 0.165 for ToppGene).

## Conclusions

brain-coX will help researchers explore candidate genes and their potential involvement in mental or neurological disorders via in silico prioritisation methods as well as allowing novel visualisation approaches. Thus, brain-coX is the ideal first step in the discovery of novel biomarkers for brain disorders or the development of new treatments for such illnesses. It is important to remember that any candidate genes prioritised by brain-coX need to be followed up to confirm their suspected involvement in the investigated brain disorder. Follow-up usually includes multiple different experimental as well as observational avenues, including, but not limited to, animal models and human iPSCs studies.

brain-coX is underpinned by the world’s largest resource of human brain microarray datasets ever assembled. By exclusively focusing on gene expression measurements in post-mortem human brain, we created a tool that is not biased by what is already known from literature or experimental approaches, but targeted to diseases originating in the brain. brain-coX also allows insights into the temporal complexity of the human brain within an easy to use web-tool. This means that brain-coX is uniquely suited towards improving our understanding of normal regulation throughout brain development. Unfortunately our effort to also make use of brain regions was thwarted due to the large inconsistencies in brain anatomical annotation between the different datasets and remains a future research goal and extension for brain-coX.

## Availability and requirements

brain-coX is available via http://shiny.bioinf.wehi.edu.au/freytag.s/. A stand-alone version is available upon request, but requires R.

## Additional files

Additional file 1: Manuscript outlining further details. (DOCX 4166 kb)

Additional file 2: List of known febrile seizure genes. (CSV 64 bytes)

Additional file 3: List of zinc transporter genes. (CSV 189 bytes)

Additional file 4: List of genes associated with epilepsy. (CSV 509 bytes)
