# Supplementary material for: brain-coX: investigating and visualising gene co-expression in seven human brain transcriptomic datasets
Source: Genome Med. 2017 Jun 8;9:55. doi: 10.1186/s13073-017-0444-y (PMC5465565; doi:10.1186/s13073-017-0444-y)
Supplement: Supplementary file 1 — Manuscript outlining further details. (DOCX 4166 kb) [file 13073_2017_444_MOESM1_ESM.docx]

**Additional Materials**

brain-coX: investigating and visualising gene co-expression in seven human brain transcriptomic datasets

Saskia Freytag^12^, Rosemary Burgess^3^, Karen L Oliver^13^ and Melanie Bahlo^124^

^1^ Population Health and Immunity Division, The Walter and Eliza Hall Institute of Medical Research, Parkville, Australia

^2^Department of Medical Biology, University of Melbourne, Parkville, Australia

^3^ Epilepsy Research Centre, Department of Medicine, Austin Health, University of Melbourne, Heidelberg, Australia

^4^Department of Mathematics and Statistics, University of Melbourne, Parkville, Australia

## RUV-corr applied to seven brain gene expression datasets

brain-coX employs the R-package RUV-corr in order to adaptively remove systematic noise from each dataset with the global version of the data-driven procedure removal of unwanted variation [1]. This step is crucial as non-biological variation is already a major source of bias in microarray experiments,. Inflated systematic noise is able to drive analysis results in prioritisation tools as gene co-expression estimates are particularly distorted [2].

In order to show that application of RUV results indeed results in more comparable datasets, we cleaned each dataset separately with both global RUV and background correction in combination with quantile normalisation. For the application of global RUV, we needed to specify a set of control genes, disease genes, candidate genes as well as the number of independent systematic noise components (k) and the value of the regularization parameter (nu). Here, we used housekeeping genes as negative control genes and defined the same disease and candidate genes as in Freytag et al [2]. The number of independent noise components and values for the regularization parameter for each dataset can be found in Additional Table 1.

After normalisation, datasets were scaled and centred before combining them. We then used a t-distributed stochastic neighbour embedding [3] (t-SNE) plot in order to visually compare normalisations (see Additional Figures 1 and 2). Note that only a set of genes common to all seven datasets was retained. It can be observed that RUV treated data displays less clustering by datasets. Furthermore, for this data the second t-SNE component can be clearly attributed to brain development; samples from early developmental periods cluster together while samples from adult periods also cluster (see Additional Figures 3 and 4). This indicates that RUV cleaning preserves change due to brain development that are of interest. Due to the unavailability of information on batches, it is not entirely clear whether RUV or conventional normalization performs better with regards to removing batch effects (see Additional Figures 5 and 6). However, the available batch information seems to indicate that clustering in the RUV normalized data is to a lesser extent due to batches than for the conventionally normalized data.

**Additional Table 1** Number of independent systematic noise components and the value of the regularization parameter for all seven datasets when using housekeeping genes as negative control genes.

| **Dataset** | **Number of independent systematic noise components** | **Value of the regularization parameter** |
| --- | --- | --- |
| Hawrylycz et al | 5 | 25000 |
| Miller et al | 4 | 500000 |
| Colantuoni et al | 3 | 15000 |
| Kang et al | 3 | 35000 |
| Hernandez et al | 1 | 0 |
| Trabzuni et al | 4 | 250000 |
| Zhang et al | 1 | 750 |

**Additional Figure 1** t-Distributed stochastic neighbour embedding [3] of data from the seven brain microarray studies detailed in Table 1 in the main paper. Different studies were treated with background correction followed by quantile-normalisation. Every point represents a sample and the colours indicate from which study a particular sample stems. Similar samples are modelled as close points while dissimilar samples are modelled as distant points.

**Additional Figure 2** t-Distributed stochastic neighbour embedding [3] of the seven brain microarray studies detailed in Table 1 in the main paper. Different studies were treated with removal of unwanted variation. Every point represents a sample and the colours indicate from which study a particular sample stems. Similar samples are modelled a close points while dissimilar samples are modelled as distant points.

**Additional Figure 3** t-Distributed stochastic neighbour embedding [3] of data from the seven brain microarray studies detailed in Table 1 in the main paper. Different studies were treated with background correction followed by quantile-normalisation. Every point represents a sample and the colours indicate the developmental period of the sample’s donor. Similar samples are modelled as close points while dissimilar samples are modelled as distant points.

**Additional Figure 4** t-Distributed stochastic neighbour embedding [3] of the seven brain microarray studies detailed in Table 1 in the main paper. Different studies were treated with removal of unwanted variation. Every point represents a sample and the colours indicate the developmental period of the sample’s donor. Similar samples are modelled a close points while dissimilar samples are modelled as distant points.

**Additional Figure 5** t-Distributed stochastic neighbour embedding [3] of the seven brain microarray studies detailed in Table 1 in the main paper. Different studies were treated with background correction followed by quantile-normalisation. Every point represents a sample and the colours indicate the batch a sample was processed in. Similar samples are modelled a close points while dissimilar samples are modelled as distant points. Note that many datasets were lacking information on batches.

**Additional Figure 6** t-Distributed stochastic neighbour embedding [3] of the seven brain microarray studies detailed in Table 1 in the main paper. Different studies were treated with removal of unwanted variation. Every point represents a sample and the colours indicate the batch a sample was processed in. Similar samples are modelled a close points while dissimilar samples are modelled as distant points. Note that many datasets were lacking information on batches.

**Prioritisation Approach**

**Algorithm 1:** brain-coX prioritisation

**1** Determination of background correlation (*K, C, R*);

**Input** : *K* is the set of known genes, *C* is the set of candidate genes, *R*

denotes all random genes

**Output**: *B* 1000 sets of background correlations

**2 repeat**

**3** Pick r of size C from R;

**4** Calculate weighted correlations of r with K;

**5 foreach** *r* **do**

**6** *Bi ←* maximum correlation with *K*;

**7 end**

**8 until** *i* = 1000;

**9** Determination of correlation threshold (*B, P* );

**Input** : *B* contains 1000 sets of background correlations, *P* is the user determined proportion of allowed associations with random genes

**Output**: *T* is the absolute correlation threshold

**10 repeat**

**11** Sort *|Bi|*;

**12 foreach** *0.05 increment j from 0 to 1* **do**

**13** *T∗ ←* value of *|Bi|* at position integer(*j×* size of *C*)

*j*

**14 end**

**15 until** *i* = 1000;

**16** Use *T∗* to estimate empirical cumulative distribution function (ECDF);

**17** *T ←* ECDF value at *P* ;

**18** Prioritisation (*K, C, T* );

**Input** : *K* is the set of known genes, *C* is the set of candidate genes, *T*

is the absolute correlation threshold

**Output**: *R* is the ranked list of prioritised genes

**19** Determine weighted correlations of *K* with *C*;

**20 foreach** *C* **do**

**21** *c ←* absolute correlations *> T* ;

**22** Sum *|c|*;

**23 if** *|c|* = 0 **then**

**24** Remove

**25 end**

**26 end**

**27** *R ←* sorted *C*

**Additional Figure 5** Pseudocode for brain-coX prioritisation approach.

**Statistical benchmarking using gene sets from KEGG and PsyGeNet**

We performed statistical benchmarking according to the leave-one-out cross-validation described in Aerts et al [4]. Hereby, we used to different sets of gene sets mined from KEGG [5] and PsyGeNet [6]. For the gene sets from KEGG, we first identified all pathways that function in the brain and then extracted their respective genes via the R-package KEGGREST. Pathways with less than 10 genes were excluded from the analysis. For the gene sets from PsyGeNet, we downloaded the entire database and also excluded diseases with less than 10 known genes. Note that statistical benchmarking was only performed for brain-coX’s default options (housekeeping genes [7], percentage threshold: 20%).

**Additional Table 2** 37 KEGG pathways and number of genes included in pathway during cross-validation

| **KEGG Identifier** | **Name** | **Number of Genes** |
| --- | --- | --- |
| hsa00010 | Glycolysis / Gluconeogenesis - Homo sapiens (human) | 58 |
| hsa00051 | Fructose and mannose metabolism - Homo sapiens (human) | 28 |
| hsa00062 | Fatty acid elongation - Homo sapiens (human) | 17 |
| hsa00071 | Fatty acid degradation - Homo sapiens (human) | 34 |
| hsa00190 | Oxidative phosphorylation - Homo sapiens (human) | 95 |
| hsa00360 | Phenylalanine metabolism - Homo sapiens (human) | 14 |
| hsa00480 | Glutathione metabolism - Homo sapiens (human) | 43 |
| hsa00500 | Starch and sucrose metabolism - Homo sapiens (human) | 36 |
| hsa00600 | Sphingolipid metabolism - Homo sapiens (human) | 39 |
| hsa00760 | Nicotinate and nicotinamide metabolism - Homo sapiens (human) | 20 |
| hsa00910 | Nitrogen metabolism - Homo sapiens (human) | 16 |
| hsa04012 | ErbB signaling pathway - Homo sapiens (human) | 81 |
| hsa04014 | Ras signaling pathway - Homo sapiens (human) | 195 |
| hsa04020 | Calcium signaling pathway - Homo sapiens (human) | 156 |
| hsa04022 | cGMP-PKG signaling pathway - Homo sapiens (human) | 147 |
| hsa04024 | cAMP signaling pathway - Homo sapiens (human) | 177 |
| hsa04068 | FoxO signaling pathway - Homo sapiens (human) | 120 |
| hsa04070 | Phosphatidylinositol signaling system - Homo sapiens (human) | 84 |
| hsa04150 | mTOR signaling pathway - Homo sapiens (human) | 52 |
| hsa04350 | TGF-beta signaling pathway - Homo sapiens (human) | 75 |
| hsa04360 | Axon guidance - Homo sapiens (human) | 117 |
| hsa04370 | VEGF signaling pathway - Homo sapiens (human) | 50 |
| hsa04720 | Long-term potentiation - Homo sapiens (human) | 62 |
| hsa04721 | Synaptic vesicle cycle - Homo sapiens (human) | 51 |
| hsa04722 | Neurotrophin signaling pathway - Homo sapiens (human) | 107 |
| hsa04723 | Retrograde endocannabinoid signaling - Homo sapiens (human) | 92 |
| hsa04724 | Glutamatergic synapse - Homo sapiens (human) | 94 |
| hsa04725 | Cholinergic synapse - Homo sapiens (human) | 103 |
| hsa04726 | Serotonergic synapse - Homo sapiens (human) | 97 |
| hsa04727 | GABAergic synapse - Homo sapiens (human) | 83 |
| hsa04728 | Dopaminergic synapse - Homo sapiens (human) | 117 |
| hsa04730 | Long-term depression - Homo sapiens (human) | 55 |
| hsa04740 | Olfactory transduction - Homo sapiens (human) | 232 |
| hsa04742 | Taste transduction - Homo sapiens (human) | 68 |
| hsa04921 | Oxytocin signaling pathway - Homo sapiens (human) | 142 |
| hsa04961 | Endocrine and other factor-regulated calcium reabsorption - Homo sapiens (human) | 45 |
| hsa04978 | Mineral absorption - Homo sapiens (human) | 47 |

**Additional Table 3** 17 psychiatric diseases and number of known genes according to PsyGeNet

| **Disease** | **Number of Genes** |
| --- | --- |
| Depression | 283 |
| Bipolar Disorder | 380 |
| Unipolar Depression | 100 |
| Mood Disorders | 127 |
| Depressive Disorder | 163 |
| Major Affective Disorder | 38 |
| Cocaine-Related Disorders | 79 |
| Alcoholism | 413 |
| Depressive Disorder | 247 |
| Suicide | 83 |
| Bipolar Depression | 10 |
| Cocaine Dependence | 21 |
| Seasonal Affective Disorder | 15 |
| Anhedonia | 17 |
| Alcohol Abuse | 50 |
| Alcoholic Intoxication | 17 |
| Binge Drinking | 10 |

**Additional Figure 6** Further accuracy measures generated from leave-one-out cross-validation using 37 KEGG pathways that function in the human brain. We also examine the effect of requiring a gene to be prioritised in multiple datasets on the accuracy measures. A) Precision of brain-coX prioritisation approach. B) Negative prediction value of the brain-coX prioritisation approach.

**Additional Figure 7** Further accuracy measures generated from leave-one-out cross-validation using 17 PsyGeNet diseases. We also examine the effect of requiring a gene to be prioritised in multiple datasets on the accuracy measures. A) Precision of brain-coX prioritisation approach. B) Negative prediction value of the brain-coX prioritisation approach.

**Additional Figure 8** Comparison of accuracy with different normalisation strategies for the 37 KEGG pathways. The red boxplots show accuracy as achieved by brain-cox’s normalisation when datasets were conventionally normalised while the blue boxplots show accuracy when the datasets were treated with RUV. A) Specificity of brain-cox’s prioritisation on all datasets. B) Sensitivity of brain-coX’s prioritisation on all datasets.

**Comparison with Weighted Gene Co-Expression Network Analysis**

Weighted gene co-expression network analysis (WGCNA) [8] is not a prioritisation approach, but aims to find modules of highly correlated genes using eigengene network methodology. Hence we defined a candidate gene as “prioritised” in the WGCNA context when it is classified with the majority of known disease genes in the same module. We tested WGCNA’s ability to distinguish between random genes and true disease genes with the help of 14 large KEGG pathways. For each pathway, we added 100 random genes. We then determined the eigengene modules on each conventionally cleaned dataset separately for these genes (with individually optimized parameters). Thus, we were able to assess whether known pathway genes were generally classified in the same module and not with the random genes by a chi-square test.

We compared this to brain-coX’s ability to prioritise any individual true pathway gene as determined by leave-one-out cross-validation described earlier with 100 random genes. This allowed us to also conduct a chi-square test assessing the ability of brain-coX to distinguish between random genes and true pathway genes. Like WGCNA, we conducted this analysis on every dataset separately.

In total, we conducted 98 tests (14 pathways x 7 datasets) for each approach. For brain-coX prioritisation, all of the 98 chi-square tests were significant (p-value <=0.05), demonstrating brain-coX’s ability to distinguish between random genes and true pathway genes. For WGCNA only 41 of the 98 chi-square tests were significant, clearly showing that this approach is not as suited towards candidate gene prioritisation.

## Case Study: Zinc transporter genes and their relationship with febrile seizures

Febrile seizures (FS) are the most common type of seizures occurring in children between the ages of 6 months and 5 years in combination with increased body temperature. Positive first-degree family history for FS increases risk of recurrence [9]. Additionally, FS have been observed to be inherited in an autosomal dominant pattern with reduced penetrance in large families [10]. This has led to several large studies in recent years trying to identify genetic factors determining FS susceptibility [11]. Despite considerable genetic heterogeneity [12], 10 genes have been securely implicated in the pathogenesis of FS (see Additional Table 4). Nevertheless, these genes only allow for an incomplete picture of the disease mechanism.

The properties of FS make the application of brain-coX particularly pertinent. The occurrence of FS in pre-school children points to the importance of brain development for this disease and its likely consequences in terms of changing gene expression, and thus co-expression, patterns. Furthermore, with the discovery of low zinc levels in children suffering from FS [13], researchers have hypothesized that zinc transporter genes are involved in the development of seizures. We used brain-coX to apply in silico prioritisation to 22 members of the two zinc transporter families *SLC30* (ZnT) and *SLC39* (ZIP) [14]. These two families regulate intracellular zinc levels, which play a key role in multiple brain functions.

Using brain-coX with individuals from the disease relevant time periods from 3 datasets (Kang et al [15], Colantuoni et al [16] and Hernandez et al datasets [17]), we found 4 genes*, SLC30A10, SLC30A9, SLC30A7* and *SLC30A3,* prioritised at a 10% threshold in at least one dataset. Apart from *SLC30A9* and *SLC30A7*, they are all predominately expressed in the brain. When we increased the threshold to 20%, we obtained 10 prioritised genes of which 3 genes (*SLC39A10*, *SLC39A12* and *SLC30A10*) were seen in more than one dataset. Interestingly, *SLC30A3* prioritised at both thresholds has been implicated in the pathogenesis of FS [18]. Note that both ToppGene and Endeavour did not rank *SLC30A3* towards the top, in case of Endeavour *SLC30A3* was ranked at the bottom with a p-value of 1.

To investigate these results further, we made use of brain-coX extensive visualizations options. In particular, we wished to assess whether there were changes in the co-expression patterns of the prioritised genes in the disease-relevant period (periods 9 and 10) in the normal brain. We would expect a gene exhibiting such changes to be a more promising candidate, as these co-regulation changes could be defective in children suffering from FS. Comparing co-expression in the disease-relevant period to co-expression in adult periods and fetal periods revealed that *SLC30A3, SLC30A10* and *SLC39A10* showed more significant changes in their regulation than any of the other candidates (see Additional Figures 8 and 9). Particularly striking is the co-expression pattern of *SLC30A3* with *GABRD* along development. *GABRD* is associated with FS [19]. This change in co-regulation intersects with the disease relevant period (see Additional Figure 11). The correlation between the expressions of these genes was positive during the fetal periods, negative during the adult periods and weak in the disease-relevant periods, which could indicate a re-setting of this pathway into its new role.

## Parameters chosen at each step for Case Study: Zinc transporter genes and their relationship with febrile seizures

Step 1: Selection of datasets

The Kang et al, Colantuoni et al and Hernandez datasets were selected for this analysis.

Step 2: Finding genes

We uploaded the known febrile seizure genes as known disease genes, the zinc transporter genes as candidate genes and genes associated with epilepsy as related disease genes. All lists are provided as additional files.

Step 3: Cleaning datasets

We chose to clean the datasets using the option Removal of Unwanted Variation with housekeeping genes as negative control genes.

Step 4: Prioritization

We selected the periods from late infancy to early childhood on which to conduct prioritization. We displayed prioritization output for all prioritized genes at 10% threshold and 20% threshold. This indicated the following genes of interest, referred to as prioritized genes of interest from here on out: *SLC30A7, SLC39A10*, *SLC39A12*, *SLC30A10, SLC30A9,* and *SLC30A3.*

Step 5: Visualization

Using the network option for the visualization (as found in the navigation bar), we plotted the networks for the known FS genes and prioritized genes of interest. To do this select all datasets, input the gene names manually in the text box provided and chose the free display option. This indicated that *SLC30A3* and *SLC30A10* are most interesting according to their topological location in the network.

Step 6: Analysis

Using the temporal option for the analysis (as found in the navigation bar), we plotted the co-expression patterns in the fetal period versus disease relevant period for the known FS genes and prioritized genes of interest. In order to this select all known FS genes separately and then input the prioritized genes of interest manually. Select periods 1-8 (embryonic to neonatal and early infancy) for the first set and periods 9-10 (late infancy to early childhood) for the second set. We repeat this analysis with the first set of periods being periods 9-10 (late infancy to early childhood) and the second set being periods 11-15 (middle and late childhood to late adulthood).

Step 7: Hot candidate

Using the analysis option for this part (as found in the navigation bar), we plotted the co-expression patterns throughout development for the known FS genes and *SLC30A3*. Simply change the candidate gene manually to *SLC30A3*.

**Additional Table 4** Genes associated with febrile seizures and their publications

| **Gene** | **Reference** |
| --- | --- |
| *SCNA1* | Escayg et al 2000, Nat Genet [20] |
| *SCN2A* | Sugawara et al 2001 PNAS [21] |
| *SCN1B* | Wallace et al 1998 Nat Genet [22] |
| *SCN9A* | Singh et al 2009 PLoS Genet [23] |
| *GABRG2* | Wallace et al 2001 Nat Genet [24] |
| *GABRD* | Dibbens et al 2004 Hum Mol Genet [19] |
| *HCN2* | Dibbens et al 2010 Ann Neurol [25] |
| *CACNA1H* | Heron et al 2007 Annals of Neurology [26] |
| *SLC12A5* | Puskarjov et al 2014 EMBO Rep [27] |
| *MASS1* | Nakayama et al 2002 Ann Neurol [28] |


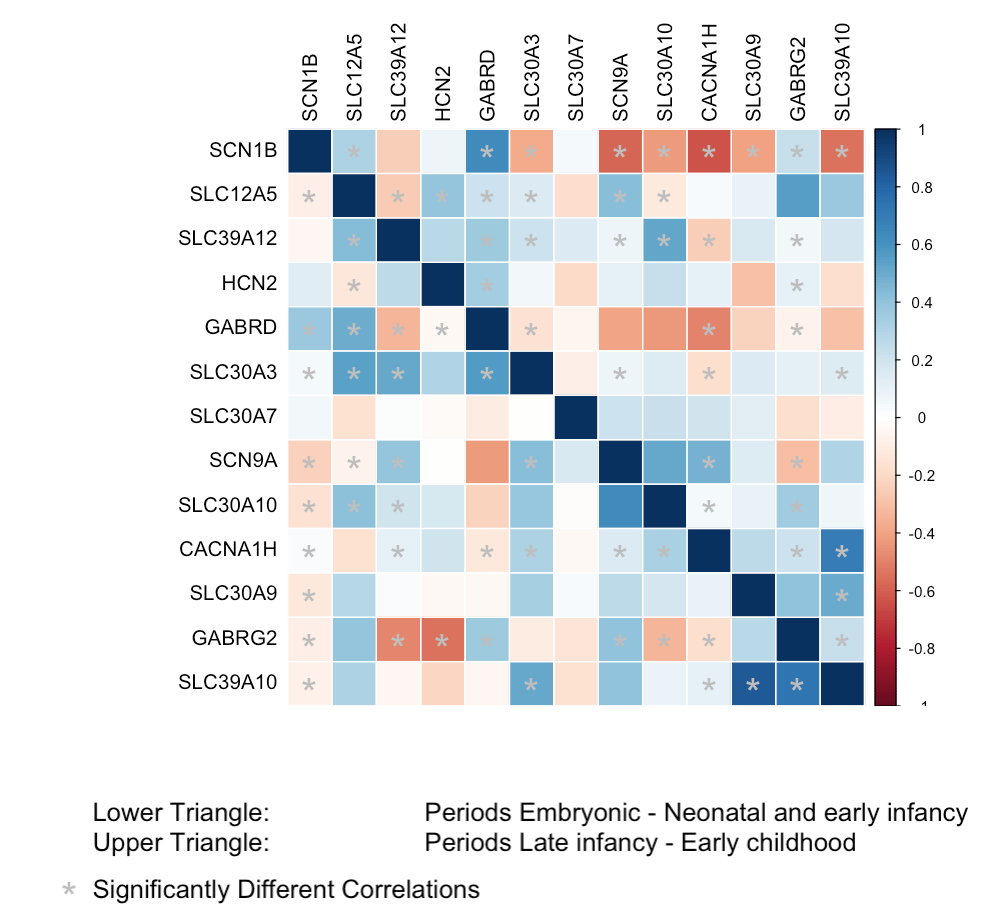


Candidates with many Co-Expression Changes Between Sets of Periods

**Additional Figure 9** Gene correlations between prioritised zinc-transporter genes and known febrile seizure genes in fetal and relevant period. Only 3 of the brain data resources were used to generate these results. The lower triangle shows gene correlation during fetal development while the upper triangle shows gene correlations during the disease relevant period. Stars mark gene correlations that are significantly different between the two investigated time periods. The green boxes highlight genes that experience the most changes with regards to their correlations with other genes across time.


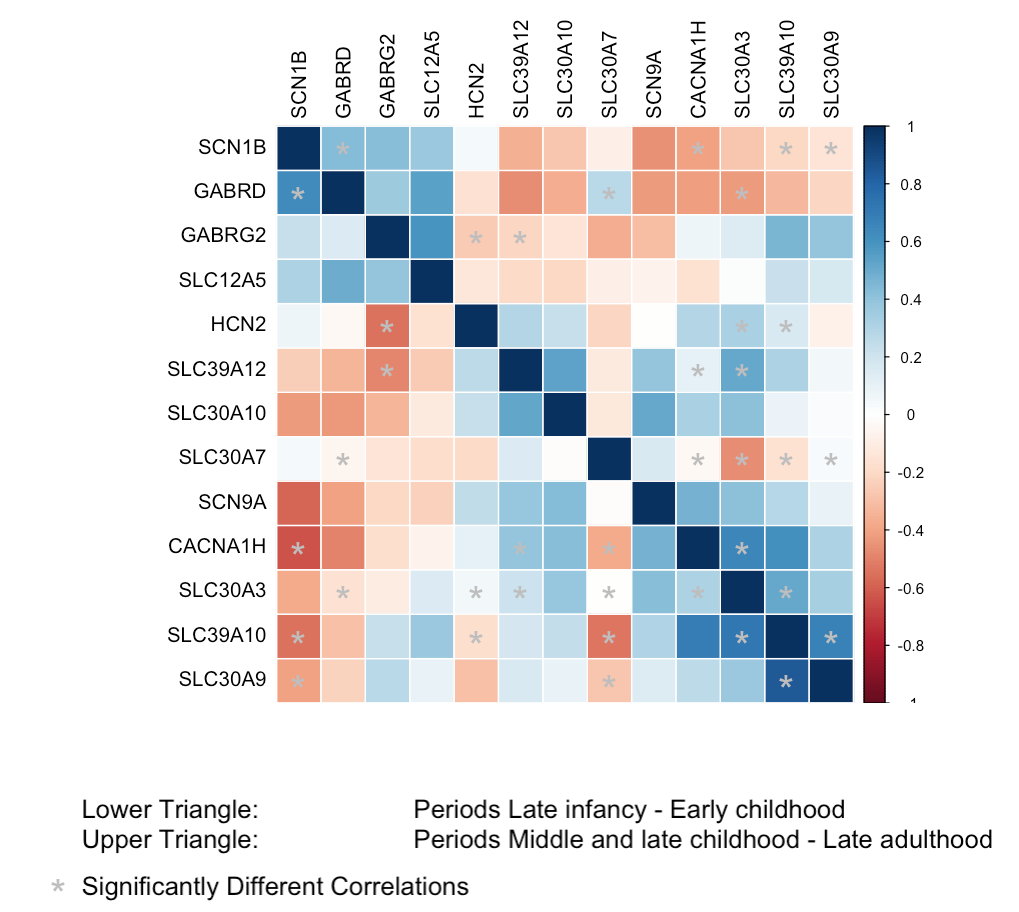


Candidates with many Co-Expression Changes Between Sets of Periods

**Additional Figure 10** Gene correlations between prioritised zinc-transporter genes and known febrile seizure genes in adult and relevant period. Only 3 of the brain data resources were used to generate these results. The upper triangle shows gene correlation during adult development while the lower triangle shows gene correlations during the disease relevant period. Stars mark gene correlations that are significantly different between the two investigated time periods. The green boxes highlight genes that experience the most changes with regards to their correlations with other genes across time.

**
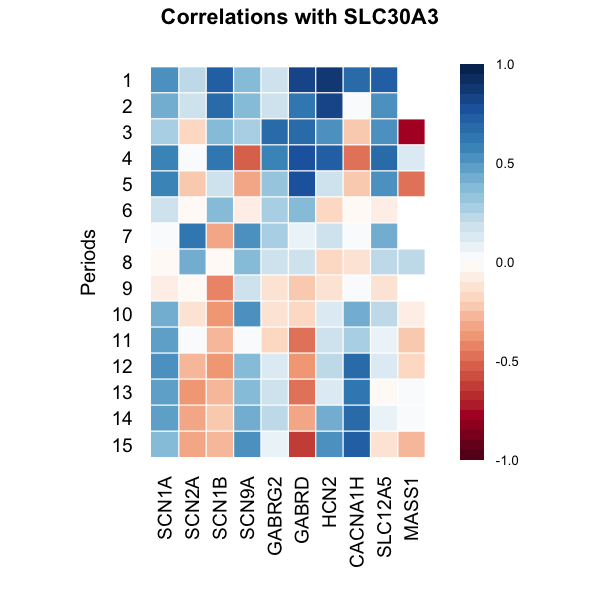
**

**Additional Figure 11** Gene correlations of SLC30A3 with known febrile seizure genes throughout different developmental periods

**References**

[1] Jacob, Laurent, Johann A. Gagnon-Bartsch, and Terence P. Speed. "Correcting gene expression data when neither the unwanted variation nor the factor of interest are observed." *Biostatistics* 17.1 (2016): 16-28.

[2] Freytag, Saskia, et al. "Systematic noise degrades gene co-expression signals but can be corrected." *BMC Bioinformatics* 16.1 (2015): 1.

[3] Van der Maaten, Laurens, and Geoffrey Hinton. "Visualizing data using t-SNE." *Journal of Machine Learning Research* 9.2579-2605 (2008): 85.

[4] Aerts, Stein, et al. "Gene prioritisation through genomic data fusion." *Nature biotechnology* 24.5 (2006): 537-544.

[5] Kanehisa, Minoru, et al. "KEGG as a reference resource for gene and protein annotation." *Nucleic acids research* 44.D1 (2016): D457-D462.

[6] Gutiérrez-Sacristán, Alba, et al. "PsyGeNET: a knowledge platform on psychiatric disorders and their genes." *Bioinformatics* (2015): btv301.

[7] Eisenberg, Eli, and Erez Y. Levanon. "Human housekeeping genes are compact." *TRENDS in Genetics* 19.7 (2003): 362-365.

[8]

[9] Van Esch, Adrianus, et al. "Antipyretic efficacy of ibuprofen and acetaminophen in children with febrile seizures." *Archives of Pediatrics & Adolescent Medicine* 149.6 (1995): 632-637.

[10] Iwasaki, Nobuaki, et al. "Molecular genetics of febrile seizures." *Epilepsia* 43.s9 (2002): 32-35.

[11] Feenstra, Bjarke, et al. "Common variants associated with general and MMR vaccine-related febrile seizures." *Nature Genetics* 46.12 (2014): 1274-1282.

[12] Racacho, Lemuel J., et al. "Evidence favoring genetic heterogeneity for febrile convulsions." *Epilepsia* 41.2 (2000): 132-139.

[13] Ganesh, R., L. Janakiraman, and B. Meenakshi. "Serum zinc levels are low in children with simple febrile seizures compared with those in children with epileptic seizures and controls." *Annals of Tropical Paediatrics* 31.4 (2011): 345-349.

[14] Marger, L., C. R. Schubert, and D. Bertrand. "Zinc: an underappreciated modulatory factor of brain function." *Biochemical pharmacology* 91.4 (2014): 426-435.

[15] Kang, Hyo Jung, et al. "Spatio-temporal transcriptome of the human brain." *Nature* 478.7370 (2011): 483-489.

[16] Colantuoni, Carlo, et al. "Temporal dynamics and genetic control of transcription in the human prefrontal cortex." *Nature* 478.7370 (2011): 519-523.

[17] Hernandez, Dena G., et al. "Integration of GWAS SNPs and tissue specific expression profiling reveal discrete eQTLs for human traits in blood and brain." *Neurobiology of disease* 47.1 (2012): 20-28.

[18] Hildebrand, Michael S., et al. "Loss of synaptic Zn2+ transporter function increases risk of febrile seizures." *Scientific Reports* 5 (2015).

[19] Dibbens, Leanne M., et al. "GABRD encoding a protein for extra-or peri-synaptic GABAA receptors is a susceptibility locus for generalized epilepsies." *Human molecular genetics* 13.13 (2004): 1315-1319.

[20] Escayg, Andrew, et al. "Mutations of SCN1A, encoding a neuronal sodium channel, in two families with GEFS+ 2." *Nature genetics* 24.4 (2000): 343-345.

[21] Sugawara, Takashi, et al. "A missense mutation of the Na+ channel αII subunit gene Nav1. 2 in a patient with febrile and afebrile seizures causes channel dysfunction." *Proceedings of the National Academy of Sciences* 98.11 (2001): 6384-6389.

[22] Wallace, Robyn H., et al. "Febrile seizures and generalized epilepsy associated with a mutation in the Na+-channel ß1 subunit gene SCN1B." *Nature genetics* 19.4 (1998): 366-370.

[23] Singh, Nanda A., et al. "A role of SCN9A in human epilepsies, as a cause of febrile seizures and as a potential modifier of Dravet syndrome." *PLoS Genet* 5.9 (2009): e1000649.

[24] Wallace, Robyn H., et al. "Mutant GABAA receptor γ2-subunit in childhood absence epilepsy and febrile seizures." *Nature genetics* 28.1 (2001): 49-52.

[25] Dibbens, Leanne M., et al. "Augmented currents of an HCN2 variant in patients with febrile seizure syndromes." *Annals of neurology* 67.4 (2010): 542-546.

[26] Heron, Sarah E., et al. "Extended spectrum of idiopathic generalized epilepsies associated with CACNA1H functional variants." *Annals of neurology* 62.6 (2007): 560-568.

[27] Puskarjov, Martin, et al. "A variant of KCC2 from patients with febrile seizures impairs neuronal Cl− extrusion and dendritic spine formation." *EMBO reports* (2014): e201438749.

[28] Nakayama, Junko, et al. "A nonsense mutation of the MASS1 gene in a family with febrile and afebrile seizures." *Annals of neurology* 52.5 (2002): 654-657.
